# SARS-CoV-2-Induced Pathology—Relevance to COVID-19 Pathophysiology

**DOI:** 10.3390/pathophysiology29020021

**Published:** 2022-06-10

**Authors:** Vsevolod A. Zinserling, Natalia Yu Semenova, Anastasia E. Bikmurzina, Natalia M. Kruglova, Oksana V. Rybalchenko, Alexander G. Markov

**Affiliations:** 1 Department of Pathology, Institute of Experimental Medicine Almazov Research Center, Saint-Petersburg 197341, Russia; natyciel87@gmail.com; 2 Center of Infectious Pathology, S.P. Botkin Infectious Hospital, Saint-Petersburg 195067, Russia; 3 Department of Physiology, Saint-Petersburg State University, Saint-Petersburg 199034, Russia; nayaspb16@gmail.com (A.E.B.); n.kruglova@spbu.ru (N.M.K.); o.rybalchenko@spbu.ru (O.V.R.); a.markov@spbu.ru (A.G.M.)

**Keywords:** viral pneumonia in a new coronavirus infection, extrapulmonary lesions, histopathology, pathogenesis, immunohistochemistry, electron microscopy

## Abstract

In spite of intensive studies of different aspects of a new coronavirus infection, many issues still remain unclear. In a screening analysis of histopathology in l200 lethal cases, authors succeeded in performing a wide spectrum of immune histochemical reactions (CD2, CD 3, CD 4, CD 5, CD 7, CD 8, CD14, CD 20, CD 31, CD 34, CD 56, CD 57, CD 68, CD 163, collagen 1,3, spike protein SARS-CoV-2, caspase-3, MLCM; ACE2 receptor, occludin, and claudin-1 and -3) and electron microscopy. The results of the histological and IHC studies of deceased people with varying degrees of severity of coronavirus infection confirmed the ability of these pathogens to cause cytoproliferative changes, primarily in epithelial and endothelial cells. Lesions of various organs are possible, while the reasons for significant differences in organotropy remain unclear. Severe respiratory failure in COVID-19 in humans is associated with a very peculiar viral pneumonia. In the pathogenesis of COVID-19, the most important role is played by lesions of the microcirculatory bed, the genesis of which requires further study, but direct viral damage is most likely. Endothelial damage can be associated with both thrombosis in vessels of various calibers, leading to characteristic complications, and the development of DIC syndrome with maximal kidney damage. Such lesions can be the basis of clinically diagnosed septic shock, while usually there are no morphological data in favor of classical sepsis caused by bacteria or fungi. A massive infiltration of the lung tissue and other organs, mainly by T lymphocytes, including those with suppressor properties, makes it necessary to conduct a differential diagnosis between the morphological manifestation of the protective cellular immune response and direct viral lesions but does not exclude the hypothesis of an immunopathological component of pathogenesis. In many of the deceased, even in the absence of clear clinical symptoms, a variety of extrapulmonary lesions were also detected. The mechanism of their development probably has a complex nature: direct lesions associated with the generalization of viral infection and vascular disorders associated with endothelial damage and having an autoimmune nature. Many aspects of the pathogenesis of coronavirus infection require further comprehensive study.

## 1. Introduction

Coronaviruses (Coronaviridae) are a large family of RNA-containing viruses that can infect both animals (their natural hosts) and humans. In humans, coronaviruses can cause a range of diseases, from mild forms of acute respiratory infection to severe acute respiratory syndrome (SARS). Over the past decades, it has been proven that coronaviruses can cause lesions of varying severity in many wild and domestic animals, causing both chronic and acute life-threatening diseases [1].

Of particular importance is the possibility of interaction of various coronaviruses with the appearance of strains with new properties [2].

Currently, four coronaviruses (HCoV 229E, OC43, NL63, and HKU1) are known to circulate in the population, which are present year-round in the structure of acute respiratory viral infections and, as a rule, cause damage to the upper respiratory tract of mild to moderate severity [3].

In 2002, a new form of zoonotic coronavirus infection causing acute respiratory syndrome (SARS) (reservoir—bats, intermediate hosts—camels) with high mortality in humans originated. In 2002–2003, an outbreak of this novel virus originating in Asia resulted in more than 8000 cases and 744 deaths in 29 countries worldwide. No cases were reported since 2004. The virus uses angiotensin-converting enzyme 2 (ACE2) for entry into host cells and infects tracheobronchial and alveolar epithelial and immune cells; other targets were not reported [4].

In two recent years, humanity has faced an infection caused by a new strain of human coronavirus (SARS-CoV-2), characterized by a variety of clinical manifestations of the disease, the lack of etiological therapy, a significant deterioration in the course of concomitant somatic pathology, and a fairly high mortality rate, which according to various studies varies widely (from 0.5 to 15%). SARS-CoV-2 is a single-stranded RNA-containing virus belonging to the family coronaviridae. The genus betacoronavirus is assigned to group 2 pathogenicity [5].

The early symptoms in men with human coronavirus consist of fever, headache, and myalgia. At days 2–7, a nonproductive cough and dyspnea are typical, which later on develop into radiographically confirmed pneumonia. Overall, lethality was reported as 10%, predominantly in persons over 60 [6,7].

The entrance gate of the pathogen is the epithelium of the upper respiratory tract and epithelial cells of the stomach and intestine. The initial stage of infection is the entry of SARS-CoV-2 into target cells that have angiotensin converting enzyme type II (ACE2) receptors. According to modern concepts, this receptor is expressed on the surface of various cells of the respiratory system, esophagus, intestines, heart, adrenal glands, bladder, brain (hypothalamus), and pituitary as well as on endothelium and macrophages [8]. The role of CD147 in SARS-CoV-2 cell invasion is also discussed [9]. The main and rapidly achievable target of SARS-CoV-2 is the type II alveolar cells (AT2) of the lungs, which determines the development of diffuse alveolar damage. With COVID-19, catarrhal gastroenterocolitis can develop as the virus affects the epithelial cells of the stomach and small and large intestine that have ACE2 receptors. The nucleocapsid protein of the virus was found in the cytoplasm of epithelial cells of the salivary glands, stomach, duodenum and rectum, and urinary tract as well as in lacrimal fluid. There is evidence of the possibility of virus detection by RNA in situ in blood vessels (endothelium), myocardium and kidneys, thyroid, esophagus, spleen, adrenals, ovary, testis, endometrium, and placenta. Although it has to be noted that the results obtained by different teams vary significantly, an optimal methodology has been proposed recently [10,11].

Direct damage to the nasal epithelium with further dissemination over nervus olfactorium through the cribriform plate can lead to brain involvement of different severities. The nasal cilia are excellent filters of particles of 5 nm. It has been established that the dissemination of SARS-CoV-2 through the systemic bloodstream or through the plate of the ethmoid bone can lead to brain damage. Changes in the sense of smell (anosmia) and taste (ageusia) in patients at an early stage of the disease may indicate both CNS damage by a virus penetrating the olfactory nerve and edema of the nasopharyngeal mucosa or viral damage to the cells of the nasal mucosa. The role of vasculitis is not excluded [12]. The virus can disseminate early and persist until the appearance of clinical symptoms: post-acute sequelae of SARS-CoV-2 (PASC) or long COVID. In the well-documented presence of virus inside the cells with light microscopy, the alterative changes may be minimal without signs of inflammation [13].

According to the pathological descriptions in the literature, at an early stage (<11 days) the disease was characterized by diffuse alveolar damage with edema, hyaline membranes, alveolar collapse, the desquamation of alveolar epithelial cells, and the appearance of scattered multinucleated giant cells of uncertain diagnostic significance [14]. The virus antigen was detected in alveolar epithelial cells and macrophages during IHC, and viral particles were detected as nucleocapsid inclusions and typical double-membrane vesicles during electron microscopy [15]. After 10–14 days, interstitial/airspace fibrosis and pneumocyte hyperplasia are described.

The changes in immunocompetent organs have not been sufficiently studied, but there is evidence that fundamental disturbances occur [16]. A number of studies postulate the leading pathogenetic role of autoimmune mechanisms based on theoretical assumptions and individual observations [17]. The main pathogenic mechanism of COVID-19 leading to patient death is severe respiratory failure, manifested in the form of acute respiratory distress syndrome (ARDS), the main morphological manifestation of which is hyaline membranes. If, at the beginning of studying the disease, the emphasis was on artificial lung ventilation in an intensive unit, similar to a severe flu (H1N1), at the moment many experts recognize this is a much less effective treatment, which has affected the change in tactics for this category of patients [18].

The most important symptom is damage to the vascular bed of different caliber vessels, leading to disorders in the blood clotting system, including lesions of the vascular endothelium and increased thrombosis from disseminated intravascular coagulation to the formation of large blood clots, including the development of thromboembolism of the main trunk and branches of the pulmonary artery with the formation of extracellular traps. The concept of immunothrombosis has also been justified. [19].

Severe manifestations of general infectious intoxication in patients with COVID-19 are due to the development of a systemic inflammatory reaction (“cytokine storm” or hyperinflammation) [20]. Previously, it was also found that the “cytokine storm” is the main factor of severe course in SARS-CoV and MERS-CoV, and increased serum IL-6 levels correlate with the development of respiratory failure, ARDS, and adverse clinical outcomes. Many studies have documented an increase in IL-6 levels in patients with COVID-19. It was found that a higher level of IL-6 is observed in patients with a more severe (complicated) course of the disease. When comparing IL-6 levels in patients with varying degrees of severity of COVID-19, it was shown that in patients with severe and extremely severe forms of the disease, the level of IL-6 in the blood serum is almost three times higher than in patients with a moderate course of the disease. The improvement of lung tissue according to computed tomography data in patients with a decrease in IL-6 levels is also described [21]. When assessing risk factors for ARDS and death, patients with ARDS had significantly higher levels of IL-6. Elevated levels of C-reactive protein (CRP), the expression of which is stimulated by IL-6, are also a biomarker of severe coronavirus infection [22]. An important role is attached to the absence of etiotropic therapy with proven effectiveness. Despite the large number of ongoing clinical studies of drug therapy of COVID-19, at present the unconditional effect of none of them has been proven. Among the drugs offered for use, there are boosted HIV protease inhibitors, inhibitors of RNA-dependent RNA polymerase of the influenza virus, interferon preparations, antimalarials, anti-COVID plasma, and anti-cytokine drugs. The effectiveness of the latter is justified by the immunosuppressive mechanism of action within the framework of the immunopathological concept of the pathogenesis of a new coronavirus infection. The use of glucocorticosteroids (dexamethasone), due to their ability to block a wide range of “inflammatory mediators”, is included in all national guidelines.

Type I interferons (IFNs) have also been shown to play an important role in the pathogenesis of COVID-19. While the rapid induction of type I IFN from the onset of the disease limits viral replication, a sustained rise in type I IFN levels in the later phase of infection is associated with aberrant inflammation and poor clinical outcomes. In one of the recent works, an alternative pathway for enhancing the production of type I IFN was established: the cyclic GMP-AMP synthase (cGAS)-stimulator of interferon genes (STING) pathway. A STING-dependent type I IFN signature was found, primarily mediated by macrophages adjacent to areas of endothelial cell injury. In addition, cGAS-STING activity was detected in lung samples from patients with COVID-19 with severe tissue destruction and an associated type I IFN response. The «lung-on-a-chip» model has shown that, in addition to macrophages, SARS-CoV-2 infection activates cGAS-STING signaling in endothelial cells through the release of mitochondrial DNA, leading to cell death and the production of type I IFN. In mice, a pharmacological inhibition of STING reduces SARS-CoV-2-induced severe lung inflammation and improves disease outcomes [23].

In the first months after the outbreak of the pandemic, autopsies in most countries of the world were either not performed at all or were performed in a very limited volume. More complete descriptions of structural changes, including the use of routine and other research methods, appeared a bit later [24]. It should be noted that there are data on the importance of postmortem morphological studies for the most correct diagnosis and, accordingly, further statistical accounting and the study of pathogenesis issues. In some European countries, clinical autopsies are carried out selectively in several universities (Germany, Switzerland, Austria, Italy, etc.) while using the most modern technologies. In others, they are either not carried out at all or are carried out in a very limited volume.

The aim of our paper was to illustrate some aspects of pathogenesis by the appropriate histopathological changes detected in our own observations.

## 2. Materials and Methods

We performed a screening analysis of 1200 autopsy cases in 2020–2021.

A clinical and morphological analysis of observations was carried out. In all cases, clinical and morphological comparisons and complete clinical autopsies were performed in the area designated for work with particularly dangerous infections, in compliance with all regulatory requirements. Pieces of all major internal organs were fixed in formalin for at least 72 h, then embedded in paraffin, and slices were stained according to generally accepted methods, including for the detection of Fe^2+^ and Fe^3+^. In some cases, immunohistochemical studies were performed using sera for CD antigens (as a marker of T cells, B cells, NK cells, and endothelial cells): CD2, CD 3, CD 4, CD 5, CD 7, CD 8, CD14, CD 20, CD 31, CD 34, CD 56, CD 57, CD 68, CD 163, and collagen 1,3 (Dako, Santa Clara, CA, USA). In addition, in the last period of time, in separate observations, it was possible to detect SARS-CoV-2 spike and nuclear protein (EpiGentek, Farmingdale, NY, USA), caspase-3 (as a marker of apoptosis; Cell Signaling Technology, Danvers, MA, USA), and MLCM (as a marker of necroptosis; Abcam, Cambridge, MA, USA) in the tissues of the lungs and a number of internal organs. As a control, four autopsy observations related to the pre-COVID period were used.

The reaction product was visualized using the Ultravision Quanto detection system HRP polymer system (Thermo Fisher Scientific, Waltham, MA, USA). The optimal conditions for the immunohistochemical reaction were selected after the procedure of staining parallel sections with negative and positive controls.

Histological preparations were studied using a Nikon ECLIPSE Ni-U microscope (Japan) with an ×10 eyepiece and ×20 and ×40 lenses. Histological preparations were analyzed in 10 fields of view at 20× magnification for each sample.

In several cases, autopsy specimens were fixed in glutaraldehyde and further embedded in Epon for electron microscopy according to the usual methods.

In several cases, the autopsy specimens were prepared for confocal microscopy with the simultaneous detection of the ACE2 receptor, occludin, and claudin-1 and -3. Human lung tissue fragments of control and research were buffered (BioVitrum, Saint Petersburg, Russia), followed by prolonged washing from the fixative, first in flowing water and then in phosphate buffer (PBS) with Ca^2+^ and Mg^2+^. Samples were passed through a series of alcohols of increasing concentrations for dehydration and encased in paraffin according to the standard procedure. Microtome-derived 5 µm thick sections were mounted on slides and dewaxed through xylene and several descending alcohols according to the standard procedure. For 30 min, the sections were heat-damaged in citrate buffer (t = 80 ℃, 0.01 M, pH = 6.0). After washing in PBS, the sections were incubated in blocking solution (0.2% Triton, 10% BSA, PBS pH = 7.4) for 2 h at 37 ℃ and then with primary rat polyclonal antibodies to claudin-1, claudin-3, and occludin (1: 200, Thermo Fisher Scientific, catalog numbers 71–7800, 34–1700, and 71–1500) and with primary mouse monoclonal antibodies to ACE-2 (1:10000, Thermo Fisher Scientific, catalog number MA5-31394) for 24 h at 4 ℃. Afterward, appropriate secondary antibodies conjugated with Alexa Fluor-488 (1:1000, Invitrogen, Waltham, MA, USA, catalog number a28175) and CF-633 (1:1000, Sigma-Aldrich, Burlington, MA, USA, catalog number SAB4600141) were applied for 120 min at 37 °C. After a series of washes in PBS, the sections were incubated in 0.1% Sudan black in 70% ethanol for 20 min to remove the nonspecific signal. Finally, after a series of washes in PBS, the nuclear dye DAPI was applied. Sections were analyzed and visualized using a Leica TCS SP5 microscope (Leica Microsystems GmbH, Wetzlar, Germany).

## 3. Results of the Study

**Figure 1:**
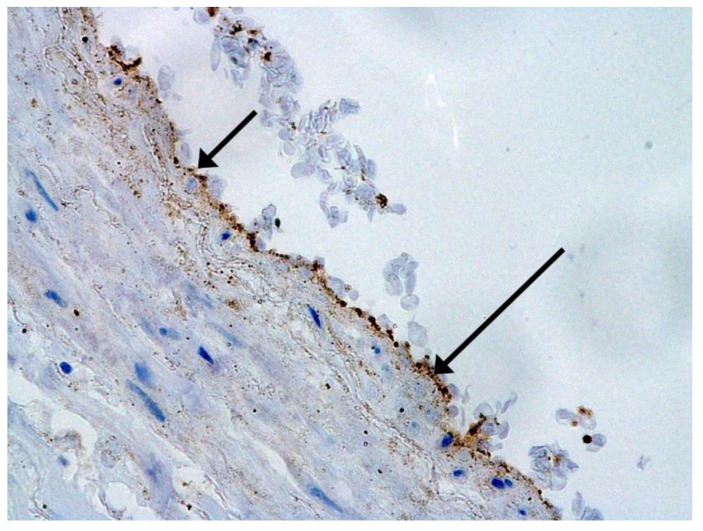
Spike antigen of SARS-CoV-2 in bronchiolar epithelium (arrow). IHC, ×100.

**Figure 2:**
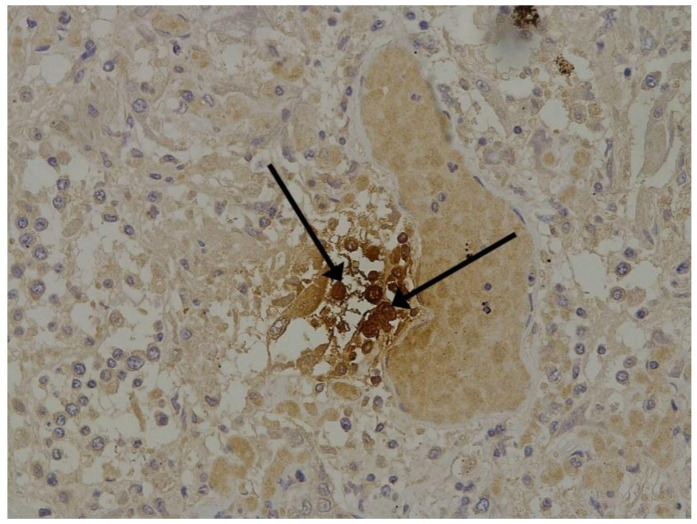
Spike antigen of SARS-CoV-2 in cytoplasm of macrophages (arrows) in lung. IHC, ×200.

We consider certain changes to be directly related to virus propagation. We succeeded in detecting virus spike antigen in the bronchiolar epithelium (Figure 1) and macrophages (Figure 2) as well.

**Figure 3:**
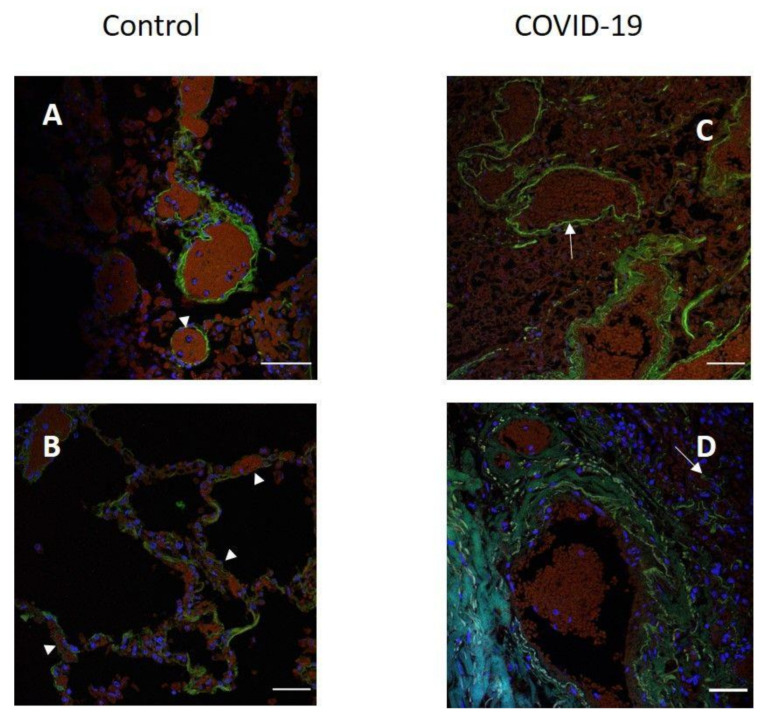
Localization of ACE2 in human lung tissue in COVID-19. Blood vessel (**A**) and alveoli (**B**) in control human lung tissue; (**C**,**D**) lung tissue in COVID-19. Green channel, ACE2; blue channel is the signal from nuclei (DAPI). Arrowhead, location of ACE2 in the control tissue and in the tissue after COVID-19. Scale bar 50 µm.

After COVID-19, lung tissue loses its architectonics. Alveolar cavities formed by alveolocytes are replaced by diffusely located cells in comparison with normal tissue (Figure 3A,B). The signal in the tissues of patients was also determined in the vascular endothelium (Figure 3C). The replacement of the alveolar tissue resulted in the signal in the lung parenchyma being defined as single dots and broken lines (Figure 3D). There is an increase in the intensity of the luminescence of antibodies to ACE2 in the tissues of patients.

**Figure 4:**
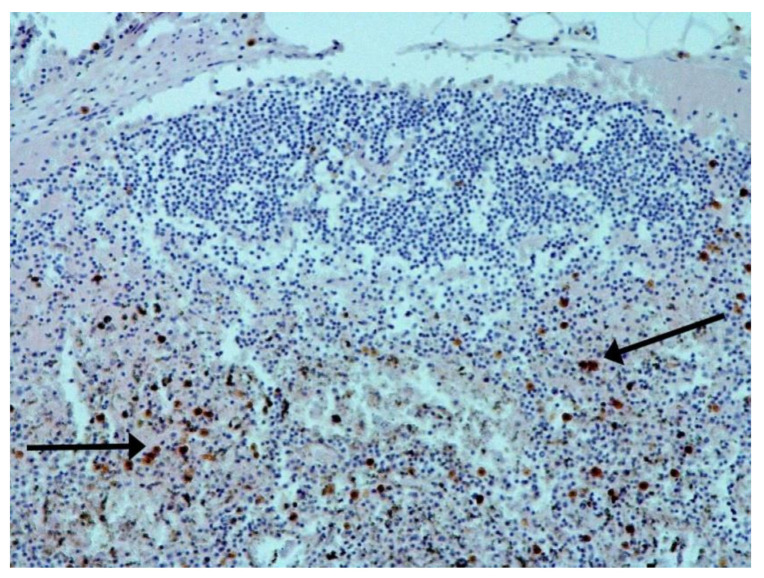
Caspase-3 (arrows) in lymph node. ICH ×100.

**Figure 5:**
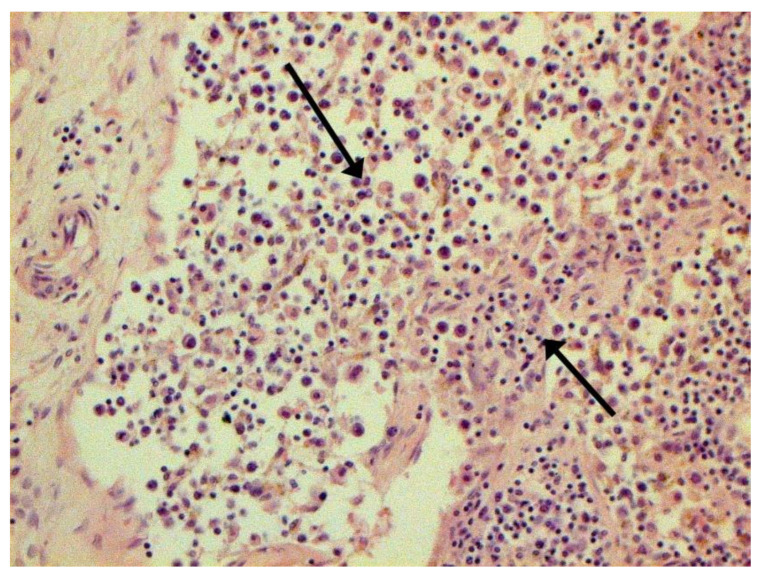
Expressed karyorrhexis (arrow) in lymph node. H-E ×200.

**Figure 6:**
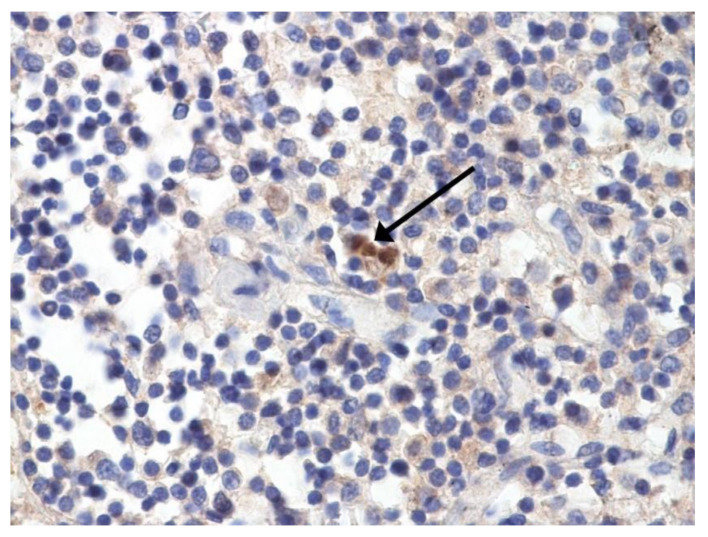
Nuclear antigen of SARS-CoV-2 (arrow) in lymph node. IHC ×400.

**Figure 7:**
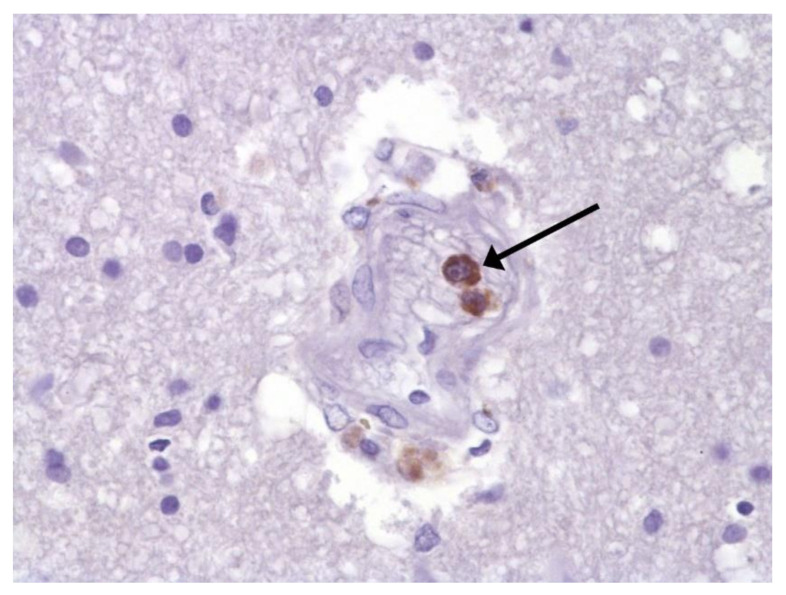
Spike protein of SARS-CoV-2 (arrow) in brain vessel. IHC ×600.

The hallmarks of apoptosis (according to expression of caspase-3) were revealed in the lungs, lymph nodules, and other organs, predominantly in the area with the small granular rhexis (Figure 4 and Figure 5.) The possibility of developing a generalized infection with damage to other organs is evident. Alterative and necrotic changes were seen in parenchymal cells. Sometimes we noted their changes in the nuclei of the cells in the lymph nodes, intestines, soft meninges, heart, pancreas, kidneys, and spleen. We succeeded in detecting spike and nuclear antigen of the virus in the lymph nodes (Figure 6), pancreas, brain (Figure 7), and adrenals. We described certain lesions in the adrenals that are probably associated with the SARS-CoV-2 virus [25].

**Figure 8:**
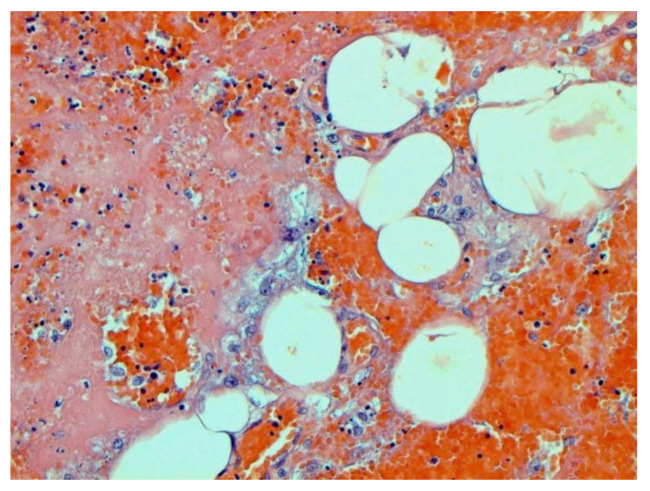
Edema and perivascular hemorrhages in lung. H-E ×100.

The main morphological substrate of respiratory insufficiency is diffuse alveolar damage. The term viral pneumonia, widely used in the clinic, essentially reflects its development. In turn, severe diffuse alveolar injury is synonymous with the clinical concept of “acute respiratory distress syndrome” (ARDS). In the pathogenesis of ARDS, without a doubt, the most important role is played by the damage to the microcirculatory bed, the genesis of which requires further study, but direct viral damage is most likely. COVID- 19 is characterized by a pronounced fullness of the capillaries of the interalveolar septa, as well as branches of the pulmonary arteries and veins, with erythrocyte sludge, fresh fibrin, organizing blood clots, and intrabronchial, intrabronchiolar, and intra-alveolar hemorrhages, which are a substrate for hemoptysis, as well as perivascular hemorrhages (Figure 8). Pronounced alveolar hemorrhagic syndrome is characteristic of most cases, up to the formation, in fact, of hemorrhagic infarcts (although true hemorrhagic infarcts are not uncommon). Pulmonary blood clots are important to distinguish from thromboembolism, as pulmonary embolism (PE) is also characteristic of COVID-19. Thrombosis of the pulmonary arteries sometimes progresses to the right parts of the heart, and thrombosis of the arteries of various organs with the development of their infarcts (myocardium, brain, intestines, kidneys, and spleen) is described. This distinguishes changes in the lungs in COVID-19 from those previously observed in influenza A/H1N1. Despite the pronounced hemorrhagic syndrome, significant deposits of hemosiderin are not observed. 

**Figure 9:**
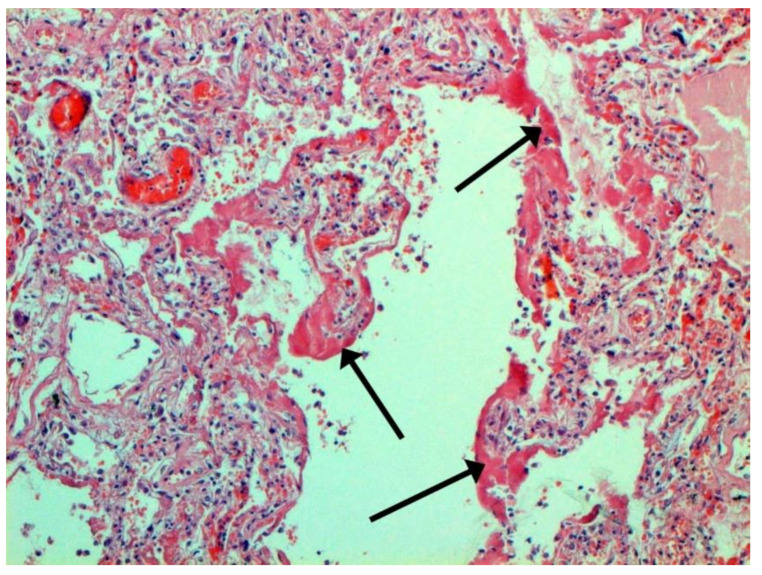
Hyaline membranes (arrows) in lung. H-E ×100.

**Figure 10:**
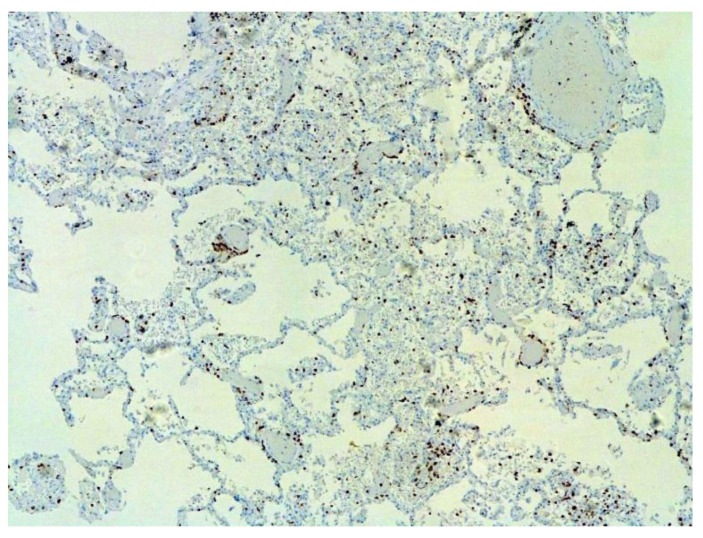
Numerous CD3+ lymphocytes in lung. IHC ×50.

**Figure 11:**
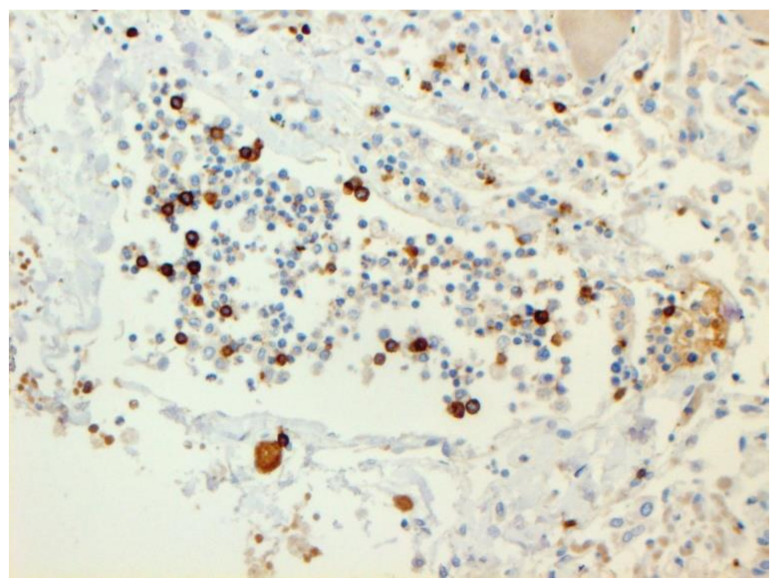
CD8+ lymphocytes in lung. IHC ×200.

**Figure 12:**
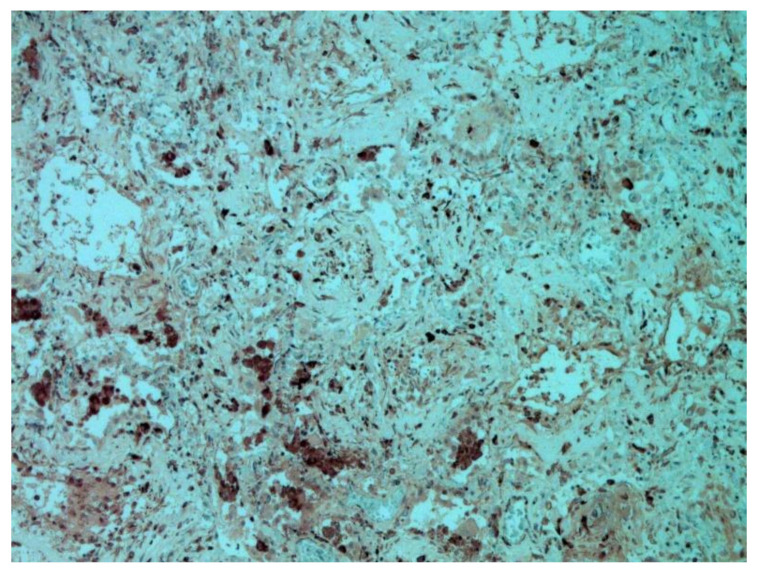
CD68+ macrophages in lung. IHC ×200.

**Figure 13:**
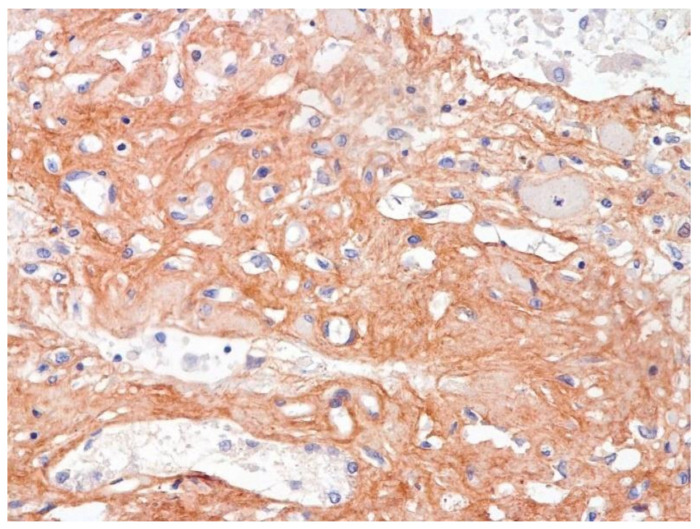
Fibrosis in lung due to collagen 3. IHC ×200.

**Figure 14:**
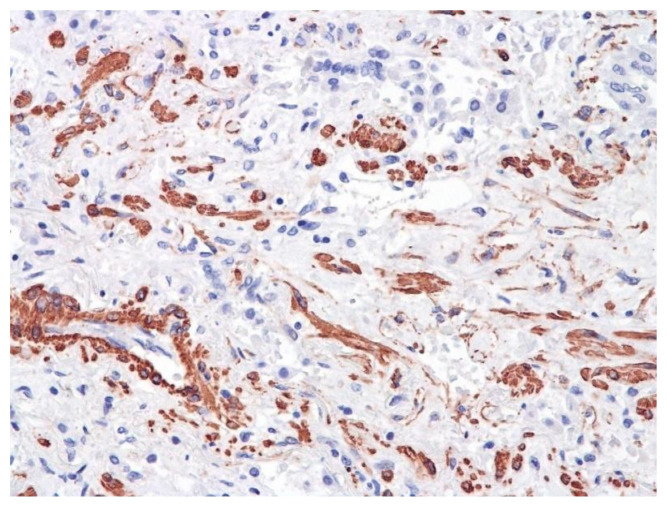
Neoangiogenesis in lung by CD34+ cells. IHC ×200.

**Figure 15:**
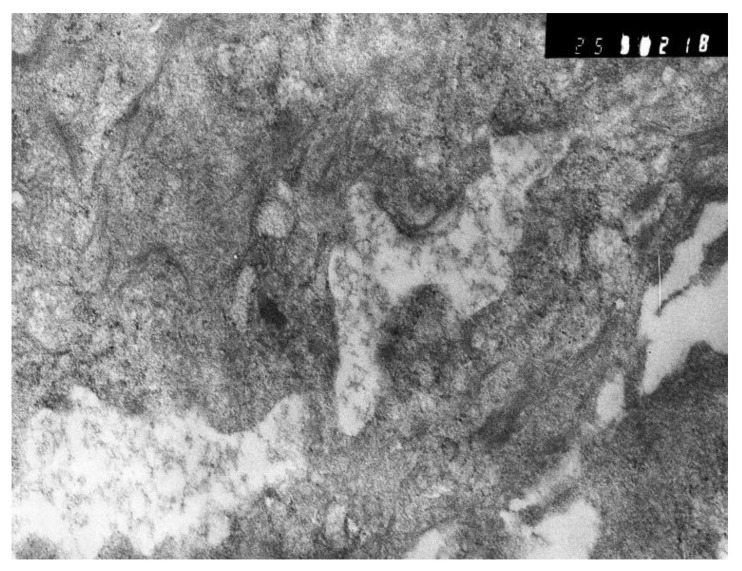
Inclusions similar to destroyed viral particle in cytoplasm of macrophage. Electron microscopy.

The dynamics of changes in ARDS associated with COVID-19 can only be judged by analogy with SARS and influenza A/H1N1pdm. In the late (productive) stage (after 7–8 days or more from the onset of the disease) of diffuse alveolar damage, macroscopically the lungs are enlarged, low-air, dense, fleshy, and can resemble the density of the liver, sometimes with diffuse whitish layers and areas of different sizes. Microscopically, siderophages, a relatively (in comparison with swine influenza) small number of hyaline membranes (Figure 9), fibrin, squamous metaplasia of the bronchial, and bronchiolar and alveolar epithelium can be detected in the lumens of the alveoli, respiratory and terminal bronchioles, the thickening of the interalveolar septa due to sclerosis, lymphoid (mostly CD3+ and CD 8+) (Figure 10 and Figure 11) and macrophage (Figure 12) infiltration, and the proliferation of type II alveolocytes. The nature of cytoproliferative changes of the epithelium in the trachea and bronchi remains unclear. In the final stage of the disease, sections of fibrous tissue may develop in all parts of the lungs (usually in the lower lobes) (Figure 13), which contributes to the development of chronic respiratory failure. It is notable that near the overgrowth of collagen fibers in the lungs, neoangiogenesis is also typical (Figure 14). The electron microscopic study revealed changed viral particles (Figure 15).

**Figure 16:**
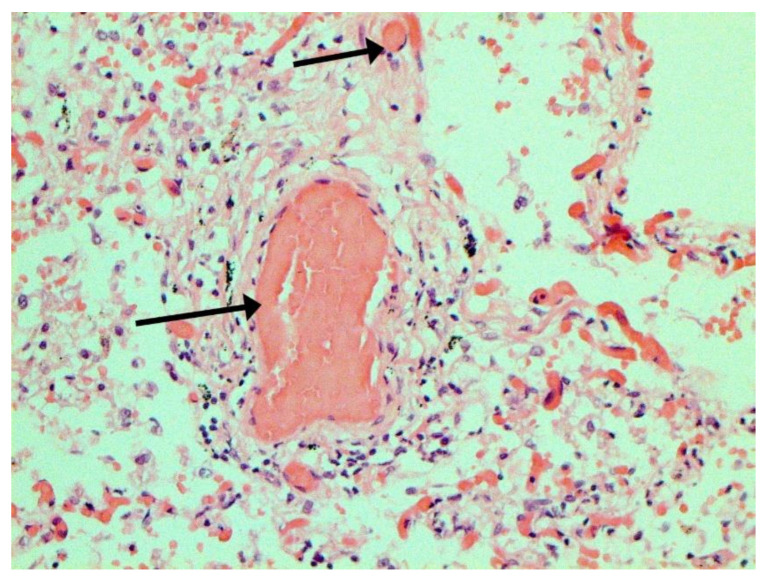
Thrombi (arrows) in lung vessels. H-E ×1500.

**Figure 17:**
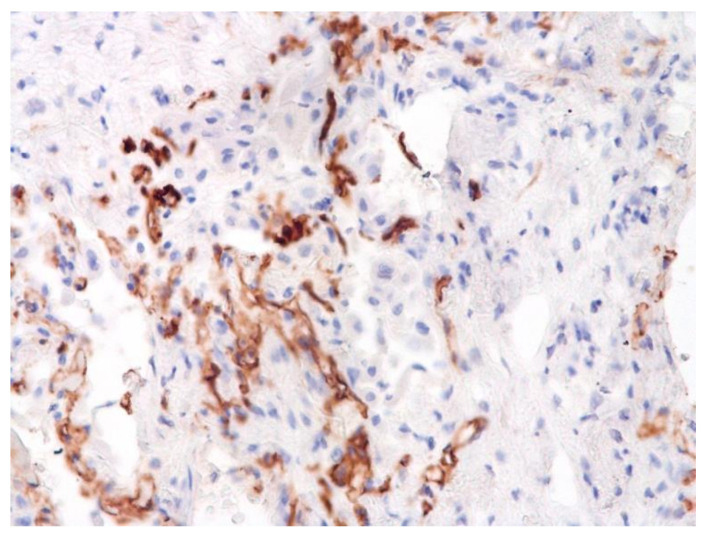
Damage of endothelium in lung vessels, CD34+ cells. ×200.

**Figure 18:**
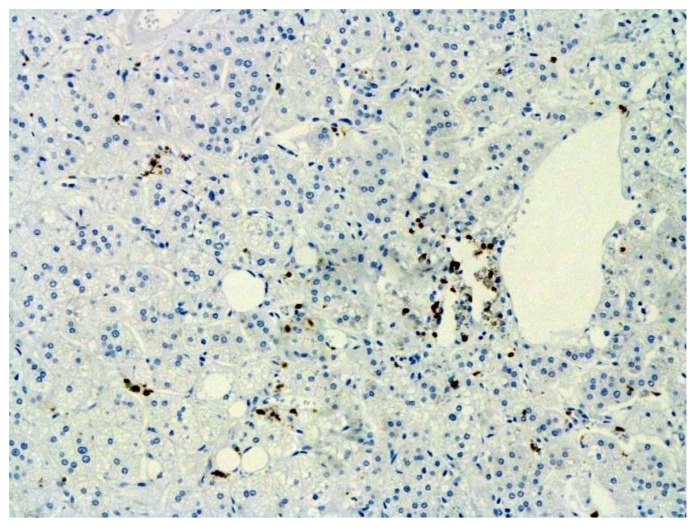
Infiltration of adrenal by CD3+ lymphocytes. IHC ×100.

**Figure 19:**
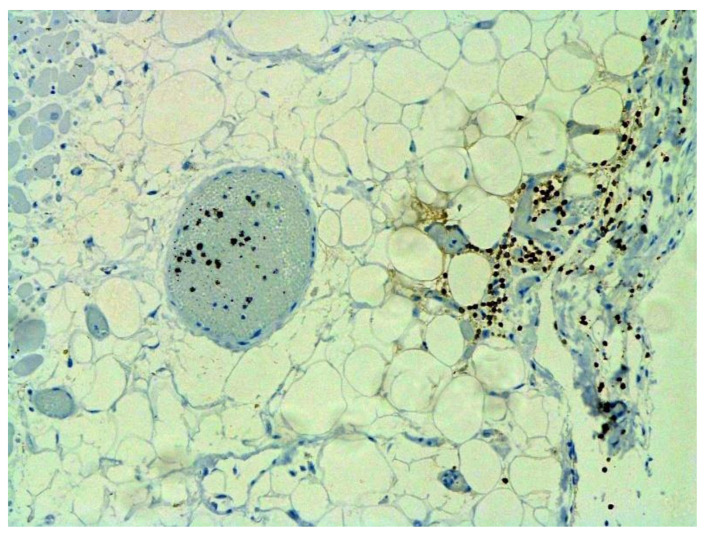
Infiltration of pericardium by CD8+ lymphocytes. IHC ×100.

**Figure 20:**
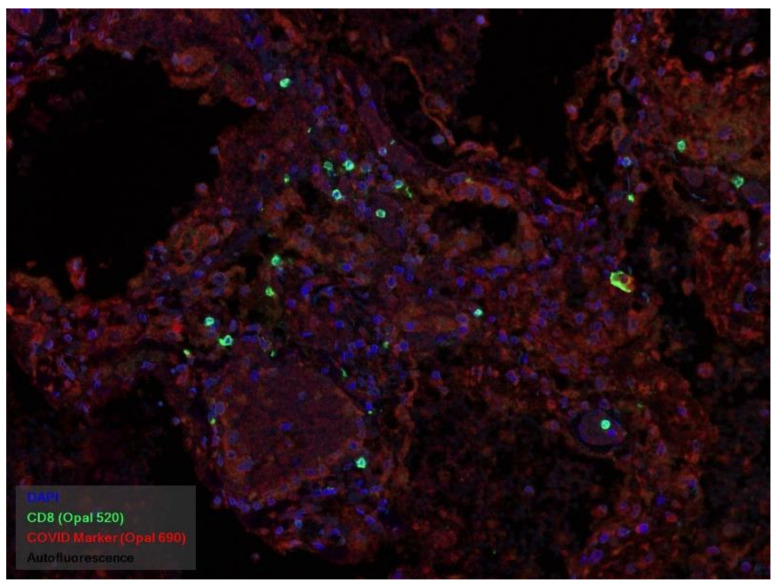
CD8+ lymphocytes (green) and spike antigen of SARS-CoV-2 (red) in lung. Luminescent microscopy, Akoya technique ×200.

In many vessels, we also observe thrombi in different vessels (Figure 16 and Figure 17). We succeeded in detecting virus spike antigen in endothelial cells as well as in other layers of vascular walls. The formation of fibrin thrombi in blood vessels (probably DIC) and infiltration by T lymphocytes, including cytotoxic cells (Figure 18 and Figure 19), were seen not only in the parenchyma of the organs but in the surrounding tissues as well. It is important to compare the localization of virus antigens and cytotoxic CD8+ cells (Figure 20).

**Figure 21:**
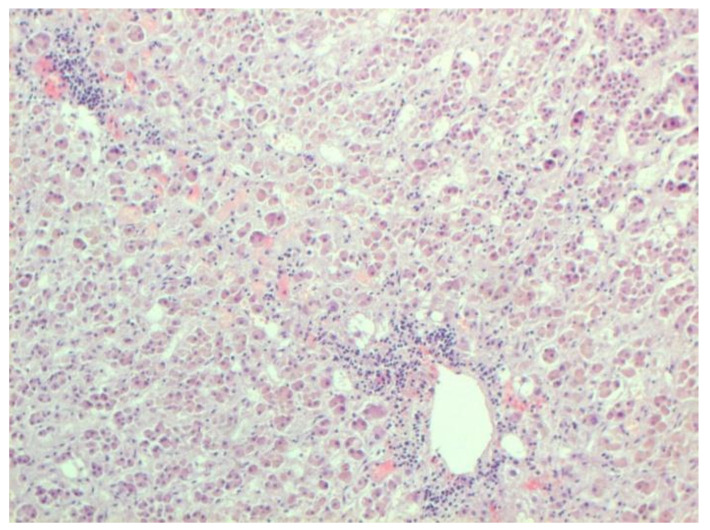
Mononuclear infiltration in adrenal. H-E ×100.

**Figure 22:**
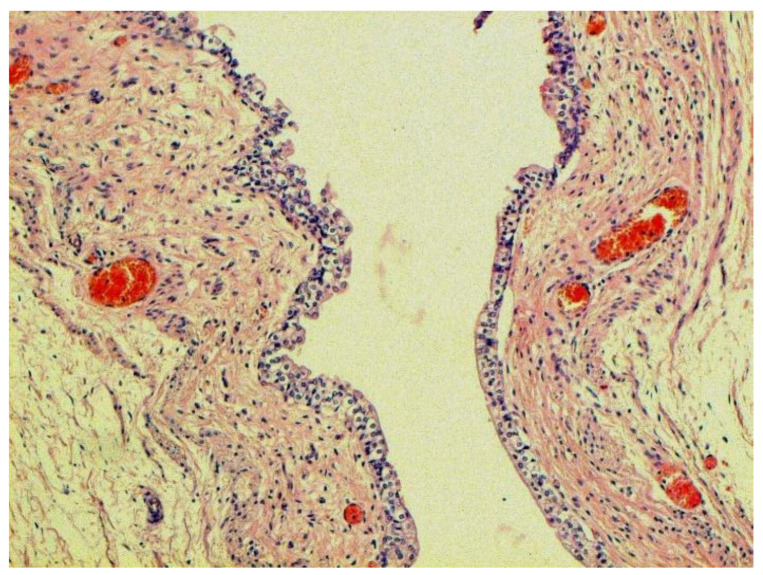
Proliferation of urethral epithelium. H-E ×100.

**Figure 23:**
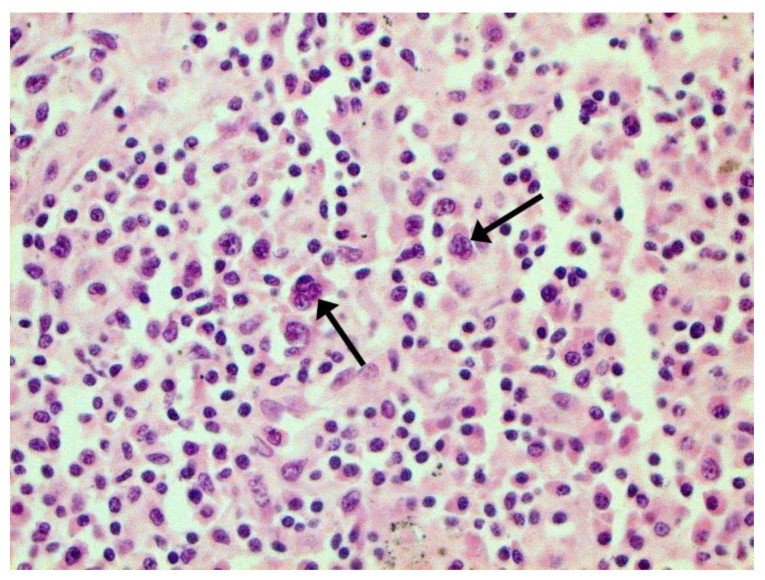
Binucleated cells with inclusions (arrows) in lymph node. H-E ×400.

**Figure 24:**
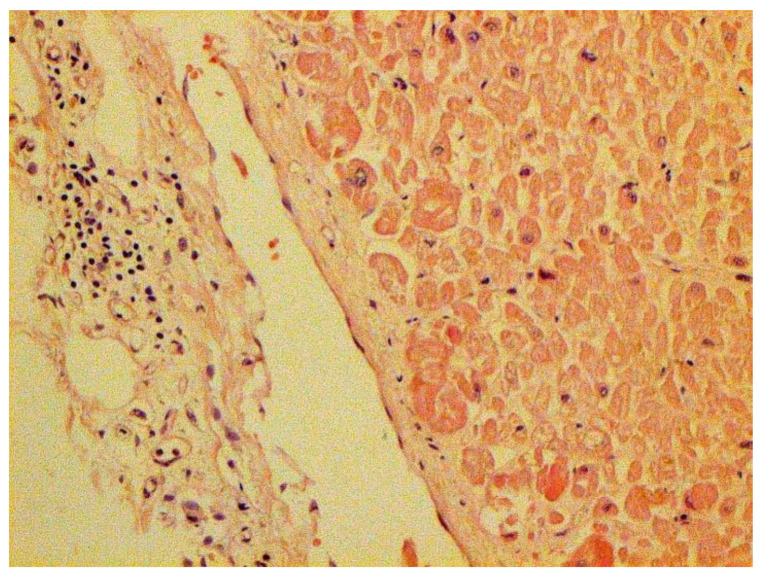
Mononuclear infiltration in capsule of kidney. H-E ×100.

**Figure 25:**
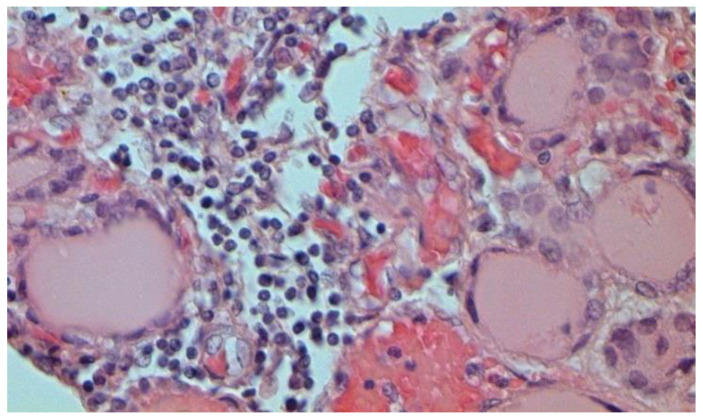
Mononuclear infiltration in thyroid gland. H-E ×400.

Certain proliferative changes occurred in cells in many internal organs and do not allow us to exclude direct viral lesions, although their nature needs further investigation (Figure 21, Figure 22, Figure 23, Figure 24 and Figure 25).

The frequency and role of joining a bacterial infection from 4 to 7 days after the onset of the disease remains unclear, which contributes to the development of viral–bacterial pneumonia, which is described mainly in the later stages of the disease. In several cases, we observed an accumulation of bacteria in the blood vessel lumen and alveoli during the histobacterioscopic investigation

There is evidence that SARS-CoV-2 is able to activate pre-existing chronic infectious processes.

In several cases where, in spite of a confirmed coronavirus infection, its role in thanatogenesis was minimal and patients died due to other severe diseases (such as ischemic heart disease, tuberculosis, HIV, chronic hepatitis in the cirrhotic stage, etc.), we still managed to observe small proliferations of bronchial epithelium. Our clinico-pathological correlations allowed us to highlight several pathogenic mechanisms in lethal cases of COVID-19:

- Generalized coronavirus infection with damage to the respiratory tract, lungs, lymph nodes, intestines, adrenal glands, and probably many other organs;
- Acute respiratory distress syndrome, practically inseparable from viral pneumonia, leading to severe respiratory failure;
- Severe vascular endothelial lesions of different calibers, causing both hemorrhages and thrombosis;
- Autoimmune damage to many organs, including leading to endocrine insufficiency;
- Septic syndrome, including acute septicemia;
- The activation of pre-existing chronic infections.

Direct causes of death, according to our experience, are:

- Acute respiratory failure;
- Acute heart failure;
- Acute renal failure;
- Septic shock;
- Intravascular disseminated coagulation syndrome (DIC);
- Multiple organ failure (dysfunction of many organs and systems);
- Secondary bacterial and fungal infections.

Our experience also allows us to conclude that statistical data related to mortality and lethality have certain subjectivity. Currently, there are no clear ideas about approaches to the statistical accounting of deaths due to coronavirus infection in the world. In some cases, the combination of typical COVID-19 clinical symptoms with a positive PCR test is considered sufficient for its registration. At the same time, it is obvious to practicing pathologists all over the world that, despite the presence of pronounced comorbid pathology in the vast majority of the deceased, the ratio of changes associated with various nosological forms can vary significantly, which justifies the need for a differentiated formulation of a post-mortem diagnosis.

When formulating a postmortem diagnosis, it is necessary to differentiate:

1. The occurrence of a fatal outcome from COVID-19 when COVID-19 is the main disease (the original cause of death);
2. The onset of death from other diseases in the presence of an infection caused by SARS-CoV-2 (diagnosed due to the detection of the SARS-CoV-2 virus by PCR) but without its clinical and morphological manifestations that could cause death. At the same time, COVID-19 can unfavorably influence the course of diseases of the circulatory system, cancer, and other diseases that cause death. In such situations, COVID-19 should not be regarded as the underlying disease (the original cause of death) and is indicated in the diagnosis as a comorbid disease. It is also necessary to analyze the possibility of developing iatrogenic complications and causes of death, primarily associated with therapy and ventilation. Among the secondary complications, pseudomembranous colitis plays a certain role.

## 4. Conclusions

The results of the histological and IHC studies of the material of deceased people with varying degrees of severity of coronavirus infection confirm the ability of these pathogens to cause cytoproliferative changes, primarily in epithelial and endothelial cells. At the same time, lesions of various organs are possible, while the reasons for significant differences in organotropy remain unclear.

Severe respiratory failure in COVID-19 in humans is associated with a very peculiar viral pneumonia. It is also quite legitimate to use the term respiratory distress syndrome (ARDS). It should be emphasized, however, that the term “non-specific” is hardly applicable to it since many of its clinical and morphological manifestations significantly distinguish it from the one we observed in swine influenza A H1N1, which does not allow us to transfer the therapeutic approaches formed in those years to a new coronavirus infection without correction.

In the pathogenesis of COVID-19, there is no doubt that the most important role is played by lesions of the microcirculatory bed, the genesis of which requires further study, but direct viral damage is most likely. Endothelial damage can be associated with both thrombosis in vessels of various calibers, leading to characteristic complications, and the development of DIC syndrome with maximal kidney damage. Such lesions can be the basis of clinically diagnosed septic shock, while there are usually no morphological data in favor of classical sepsis caused by bacteria or fungi, although we have found signs of bacteremia with an intravascular accumulation of microorganisms in separate observations. There were no signs of mycotic lesions on our material. The frequency and role of superinfection from 4-7 days after the onset of the disease remains unclear, which contributes to the development of viral–bacterial pneumonia, which is described mainly in later stages of the disease, including with a ventilator-associated mechanism. It should be noted that in observations following long-term antibiotic therapy, postmortem autopsy revealed pseudomembranous colitis in which a significant role was taken by Clostridium difficile.

A massive infiltration of lung tissue and other organs, mainly by T lymphocytes, including those with suppressor properties, makes it necessary to conduct a differential diagnosis between the morphological manifestation of the protective cellular immune response and direct viral lesions but does not exclude the hypothesis of an immunopathological component of pathogenesis. Currently, the morphological work in which such a diagnosis was carried out is unknown. The presence of false positive reactions when using a number of both poly-and monoclonal sera can be presumably associated with the presence of numerous intersections between SARS-CoV-2 and human antigens.

To assess the possible consequences of the identified lesions in surviving patients, catamnestic observation is certainly required, but judging by the literature data, the formation of fibrosis is very likely, the degree of reversibility of which is still impossible to judge. A wide variety of clinical and neurological syndromes are described, the genesis of which remains undeciphered. It is also necessary to take into account the emerging data on the possibility of preserving the virus in the body after clinical recovery [13]. It should also be remembered that coronaviruses have the ability of to cause chronic lesions in a number of animals with the development of granulomatous changes in them, which, however, have not yet been described in humans [26].

In many of the deceased, even in the absence of clear clinical symptoms, a variety of extrapulmonary lesions are also detected. The mechanism of their development probably has a complex nature: direct lesions associated with the generalization of viral infection and vascular disorders associated with endothelial damage and having an autoimmune nature. Many aspects of the pathogenesis of coronavirus infection require further comprehensive study, including clinicians, virologists, radiologists, immunologists, pharmacologists, and pathologists. It is possible that detailed histotopograpic studies (currently not provided) can allow us to obtain more information concerning the methods of virus dissemination.

## Data Availability

Results of postmortem studies are not accessible in open databases.
